# Intraocular Lens Power Calculation after Phototherapeutic Keratectomy: Case Report and a New Method

**DOI:** 10.4103/0974-9233.53373

**Published:** 2008

**Authors:** Omar Kirat

**Affiliations:** From Anterior Segment Division, King Khaled Eye Specialist Hospital, Riyadh, Kingdom of Saudi Arabia

**Keywords:** IOL power calculation, PTK, refractive surprise, keratometry, formula

## Abstract

To report a case of cataract extraction and intraocular lens (IOL) implantation after phototherapeutic keratectomy (PTK). The IOL power was calculated using the single-K and the double-K SRK/T formula, as well as the Haigis formula after modifying the post PTK corneal power using methods described for corneal power measurements after myopic excimer laser treatment (photorefractive keratectomy (PRK) and LASIK). A new method for IOL power calculation after PTK is introduced.

Excimer laser alters the anterior corneal surface in a way that makes subsequent corneal power measurements using keratometry or topography inaccurate.^1^ The corneal power may be overestimated after excimer laser procedures that correct myopia (PRK, LASIK and LASEK) and this can lead to a hyperopic refractive surprise after cataract extraction.^2^

The effect of PTK on IOL calculation has not been studied in great details – compared to that of myopia-treating PRK or LASIK, although PTK induces a hyperopic shift in most of the cases especially with deeper ablations,^3^ and can be considered as an hyperopic treatment of some sort. this article demonstrates the effect of PTK on subsequent IOL power calculation, introduces some methods to improve the accuracy of IOL power calculation and introduces a new method for IOL power calculation after PTK.

## Case Report

A 77-year-old male presented to the Anterior Segment Clinic of the King Khaled Eye Specialist Hospital with significant smooth climatic droplet keratopathy, Salzmann's nodules and immature cataract in the right eye. Both eyes had glaucoma controlled on one medication, the left eye was pseudophakic. The patient's uncorrected visual acuity (UCVA) was 20/100 in the right and 20/400 in the left eye. His best spectacle corrected visual acuity (BSCVA) was 20/80 in the right and 20/400 in the left eye. Refraction was +7.00 -4.5 × 100 and +1.25 – 1.50 × 90 in the right and left eye, respectively.

To improve vision in the right eye, it was decided to perform PTK first and then cataract extraction and intraocular lens implantation. Pre-PTK IOL calculation was done, the average keratometric measurement before PTK was 43.62 diopters, the axial length was 22.58 mm, and the IOL power, estimated for emmetropia using SRK/T and Haigis formulas with an A constant of 118.9, was 24.01 D and 25.59 D respectively. His average pre-PTK central corneal thickness, measured with ultrasound pachymetry, was 580 microns. The treatment plan was explained to the patient and an informed consent was obtained prior to all the procedures. He then underwent superficial keratectomy to remove Salzmann's nodules, plus PTK, in a stop-and-check manner using the Nidek EC-5000 machine. Balanced salt solution was used as a masking fluid. The total ablation depth entered into the machine was 200 microns, the treatment zone was 7.5 mm. No anti-hyperopia treatment was done. At the end of the procedure, Mitomycin C was applied in a concentration of 0.02mg/ml for 45 seconds and then was washed off. After the PTK, the corneal clarity improved dramatically with only a faint residual scar remaining. Eight months later his post PTK central corneal thickness was 509 microns with an actual net central ablation depth of 72 microns. Post PTK axial length was 22.38 mm, the average post PTK keratometry was 40.63 D. The IOL power was calculated using the Haigis formula and the single and double-K^4^ SRK/T formula. In the double-K method the average pre-PTK corneal power was used to calculate the effective lens position (ELP). The average post PTK corneal power was used, with and without modification, to calculate the IOL power in the single- as well as the double-K method (see Table).

**Table 1:** IOL Calculation was Based on an A-Constant of 118.9. Maloney's Method: Corneal Power = Keratometry X 1.11 – 4.9; Modified Maloney's Method: Corneal Power = Keratometry X 1.11 -6.1; Shammas Method: Corneal Power = Keratometry X 1.143 – 6.8; Double-K SRK/T Is Not Applicable Before PTK

Emmetropia IOL				
			Keratometry	
Haigis	Double-K SRK/T	Single-K SRK/T		
25.59D	Not applicable	24.01D	43.62	Pre PTK

30.59D	28.82D	27.71D	40.63D	Unmodified post PTK

31.20D	29.42D	28.15D	40.19D	Maloney's method

32.84D*	31.05D	29.34D	38.99	Modified Maloney's method

31.95D	30.17D	28.7D	39.64	Shammas method

* The highest IOL power was obtained using the modified Maloney's method and Haigis formula

The lowest value of the modified average corneal power was 38.99 D as obtained with the modified Maloney's method.^5^ Using this corneal power with Haigis formula resulted in the highest IOL power for emmetropia (32.84D).

The patient underwent uneventful phacoemulsification with posterior chamber IOL implantation. The implanted IOL power was 33 D, single-piece from Acrysoft Alcon Laboratories, A-constant 118.4 with an expected postoperative refraction of -0.48 D. Six weeks later, the patient's uncorrected visual acuity (UCVA) was 20/50, and his BSCVA was 20/40 with the refraction of +1.00 -3.00×150, the spherical equivalent (SE) was -0.5 D which almost equals to what was anticipated by the Haigis formula.

## Discussion

Many patients present with significant corneal opacity and cataract which necessitate treating both conditions – in order to achieve good post operative visual acuity. And while performing accurate keratometry on a cornea with smooth superficial opacities is possible, the cataract surgery itself can be difficult and probably risky. The implanted intraocular lens may not improve the visual acuity to the level that satisfies the patient and/or the surgeon due to the presence of the corneal opacity. On the other hand, performing cataract surgery after PTK is easier than before it, but the IOL power is expected to be underestimated due to error in the intraocular lens power calculation.

To calculate the IOL power of this patient, some of the methods, originally described, to calculate the IOL power after myopic excimer laser treatment were used. The methods that depend on spherical equivalent change were excluded. To calculate the post PTK corneal power, the pre PTK corneal power measurements were considered accurate as long as there is no distortion of the mires, and were used to calculate the effective lens position (ELP) in the double-K method. Corneal power measured with standard keratometry after PTK (average K post-PTK= 40.63.) was considered as an overestimated value and therefore was modified and then used to calculate the IOL power in Haigis formula and in the double-K SRK/T formula. Although Maloney's method^5^ was designed to be used after myopic excimer laser utilizing central topographic measurements, it was used for the first time on this patient to adjust keratometric rather than topographic values, and also for the first time after PTK. This resulted in a lower corneal power measurement (average K = 40.19), the modified Maloney's method^5^ gave the lowest corneal power(average K=38.99), and Shammas method^6^ (average K= 39.64) was in between. The IOL power was then calculated using the SRK/T formula in a single and a double-K manner and the Haigis formula. The highest power was obtained using the Haigis formula with the modified Maloney's method with a stable post operative SE of -0.5D. The modified Maloney's method calculated the correct post-PTK corneal power in this patient (38.99D). When using this corneal power in the single- or even the double-K SRK/T formula the calculated IOL power was weaker and would have resulted in a hyperopic surprise (see Table). Using this emmetropia IOL value, the correct average post PTK corneal power was back calculated and was found to be 36.9 D for the double-K SRK/T formula. On the other hand, using this value in the Haigis formula would have resulted in an IOL power of 35.65D for emmetropia. This significant difference in the IOL power between the double-K SRK/T and the Haigis formula is probably due to the decreased accuracy of the SRK/T formula in hyperopic eyes.^7^

In general, the Haigis formula was better than the double-K SRK/T formula in this patient, the least accurate was the single-K SRK/T formula. This case demonstrate the hyperopic effect of PTK and it's impact on subsequent IOL calculation. However, this conclusion is limited as it is based on the findings in one patient only, and, therefore, many cases need to be done in order to favor one modified keratometric value over the other, or one formula over the other.

The following method is proposed to calculate post PTK corneal power: Obtain pre PTK corneal power, and central corneal ultrasound pachymetry, convert the change in corneal thickness induced by PTK into diopters and subtract it from the pre-PTK corneal power to get the post PTK corneal power. The resultant post PTK corneal power can then be used to calculate the IOL power using a formula that dose not need the keratometric value to calculate the ELP (e.g. the Haigis formula), or can be used together with the pre PTK corneal power in a double-K manner. Although the Munnerlyn formula^8^ can, at least theoretically, convert ablation depth into diopters, many patients are needed to calculate how many microns of corneal tissue ablated with PTK that correspond to a one diopter of hyperopic shift for a given treatment zone and a given formula. In this patient the change in ablation depth was 72 microns. This will result in one diopter of hyperopia for each 10.71 microns ablated in the case of the double-K SRK/T formula. And one diopter of hyperopia for each 15.5 mircons of ablation in the case of Haigis formula.

Converting the ablation depth entered into the excimer machine at the time of performing the PTK into diopters can overestimate the hyperopic shift as the actual ablation is usually less than that entered into the machine due to the use of masking fluid, and the fact that post PTK healing will decrease the induced hyperopic shift^3^ as evident in this patient. The most possible accurate method is to use the stable post PTK central corneal thickness, as the central cornea is the most important factor when considering the Stiles Crowfordt effect.^9^ This method needs to be tested on a large number of patients and if proven accurate may help in calculating the IOL power after PTK.
